# SUMOylation-Mediated Response to Mitochondrial Stress

**DOI:** 10.3390/ijms21165657

**Published:** 2020-08-06

**Authors:** Jianli He, Jinke Cheng, Tianshi Wang

**Affiliations:** 1 Department of Biochemistry and Molecular Cell Biology, Shanghai Key Laboratory for Tumor Microenvironment and Inflammation, Shanghai Jiao Tong University School of Medicine, Shanghai 200025, China; jianlihe@shsmu.edu.cn; 2 Key Laboratory of Cell Differentiation and Apoptosis of Chinese Ministry of Education, Shanghai Jiao Tong University School of Medicine, Shanghai 200025, China

**Keywords:** SUMOylation, SUMO, SENPs, mitochondrion, stress response

## Abstract

Mitochondrial stress is considered as a factor that reprograms the mitochondrial biogenesis and metabolism. As known, SUMOylation occurs through a series of stress-induced biochemical reactions. During the process of SUMOylation, the small ubiquitin-like modifier (SUMO) and its specific proteases (SENPs) are key signal molecules. Furthermore, they are considered as novel mitochondrial stress sensors that respond to the signals produced by various stresses. The responses are critical for mitochondrial homeostasis. The scope of this review is to provide an overview of the function of SUMOylation in the mitochondrial stress response, to delineate a SUMOylation-involved signal network diagram, and to highlight a number of key questions that remain answered.

## 1. Introduction

### 1.1. Mitochondrial Stress Response

Mitochondrial health is of vital importance. In the course of a life, mitochondria produce energy through the metabolism of small molecules, such as glucose, fatty acids, and amino acids [1,2,3]. During this process, intermediates produced from the mitochondrial metabolism can be used as raw materials for anabolism and signal molecules that regulate cell activity [4]. Increasing numbers of studies are providing evidence to validate the claim that the metabolic activities and functions of mitochondria are triggered by extracellular environmental alterations [5,6,7,8]. The environmental alterations include oxygen, temperature, nutrients, endocrine, toxicity and other cellular stress-induced factors [5,6,7,8]. The response of mitochondria to these factors is called the mitochondrial stress response.

The levels of the mitochondrial stress response reflect mitochondrial adaptations, including those of morphology and functions. Generally, excess nutrition can induce an increase in fat synthesis in the body and a decrease in fatty acid oxidative decomposition in mitochondria. A low level of mitochondrial stress response leads to some metabolic syndromes including obesity, hyperglycemia, hypertension, dyslipidemia, etc. [9]. Enhanced calorie restriction or exercise increase mitochondrial fatty acid oxidation, which in turn reduces insulin resistance and improves the mitochondrial adaptation to stress [10]. The cellular signaling pathways that regulate mitochondrial stress response are still poorly understood.

### 1.2. SUMOylation is a Stress-Induced Process

**Figure 1:**
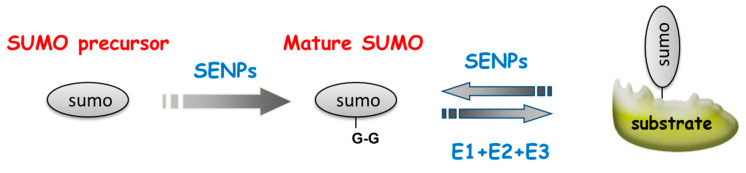
The mechanism of small ubiquitin-related modifier (SUMO)ylation. SUMO-specific proteases (SENPs) cleave the C-terminus of SUMO to expose G-G motif and interact with SUMO activating enzyme (E1), and E1 transfers SUMO to ubiquitin conjugating enzyme (E2). Then, E2 conjugates C terminus of SUMO and lysine residues of SUMO targets. At times, a specific SUMO ligase (E3) may increase SUMOylation specificity and efficiency.

Small ubiquitin-related modifier (SUMO) proteins were discovered in the 1990s [11]. Despite the fact that SUMO only has ~18% sequence identity with ubiquitin, they become functional mature proteins via a similar mechanism. The globular β-grasp fold and C-terminal diglycine (G-G) motif of SUMO can bind the lysine residue of targets (Figure 1) [12]. Based on current research, mammalian SUMO proteins have the following features: (1) there are four isoforms (SUMO1–4); (2) human SUMO1–3 are widely distributes in tissues but SUMO4 is specifically expressed in the kidneys, lymph nodes and spleen [13]; (3) SUMO2 has 95% similarity with SUMO3 but is only 50% identical to SUMO1 [14]; and (4) SUMO1–3 harbor internal SUMOylation sites at their lysine 11 (K11) to form poly-SUMO chains [15]. The studies concerning SUMO illustrate that it is very crucial for the stress response in eukaryotes [16,17,18,19].

As shown in Figure 1, SUMOylation is a revisable and dynamic protein post-translational modification. Initially, the endopeptidases of SUMO-specific proteases (SENPs) cleave the C-terminus of SUMO to expose the SUMO-interacting motif G-G for conjugation. The activator of SUMO (SAE1, AOS1) called E1 promotes the adenylation of the exposed C-terminus to form the SUMO–adenosine monophosphate (AMP) intermediate; then, SUMO is transferred to ubiquitin-like modifier activating enzyme 2 (SAE2, UBA2) to form a thioester bond. SAE transfers SUMO to E2, ubiquitin conjugating enzyme 9 (UBC9), and forms an isopeptide bond between SUMO C-terminus and lysine residues of its substrate via E2 conjugation; a specific E3 ligase may contribute to this process [20]. Mitochondrial E3 ubiquitin ligase (MAPL) has been identified as a mitochondrial SUMO E3 ligase [21]. The RING (really interesting new gene) domain of MAPL is exposed to the cytoplasm and SUMOylates certain proteins, such as dynamin-related protein (DRP1) [22]. Finally, SENPs remove SUMO from their substrates [23]. Notably, the SUMOylation levels of the proteins are varied in response to mitochondrial stress [24]. As SUMO deconjugation proteases, SENPs have been considered to be the regulators of SUMOylation upon cellular stresses [25,26,27,28].

Extra- and intra-cellular stimuli such as the nutritional state, redox state and ions state can trigger the occurrence of SUMOylation. For instance, activity-dependent calcium concentration induces dephosphorylation by calcineurin and then switches from SUMOylation to acetylation for myocyte enhancer factor 2A (MEF2A) [29]. The cellular senescence is considered to be chronic oxidative stress for mitochondria; similar to the ubiquitinated protein, the amount of SUMOylated protein in rodent tissues increases with age independently of calorie restriction [30]. SUMO expression has been shown to increase upon hypoxia; despite SUMO1-p53 being unaffected, SUMO2/3-modified p53 increases under oxidative stress [31]. Global mitochondrial SUMOylation detection illustrates that calorie restriction can decrease the mitochondrial pan-SUMOylation, which is consistent with pan-acetylation [24]. Overall, all evidence suggests that SUMOylation is a stress-induced post-translational modification.

## 2. Roles of SUMOylation in Mitochondrial Biogenesis

**Figure 2:**
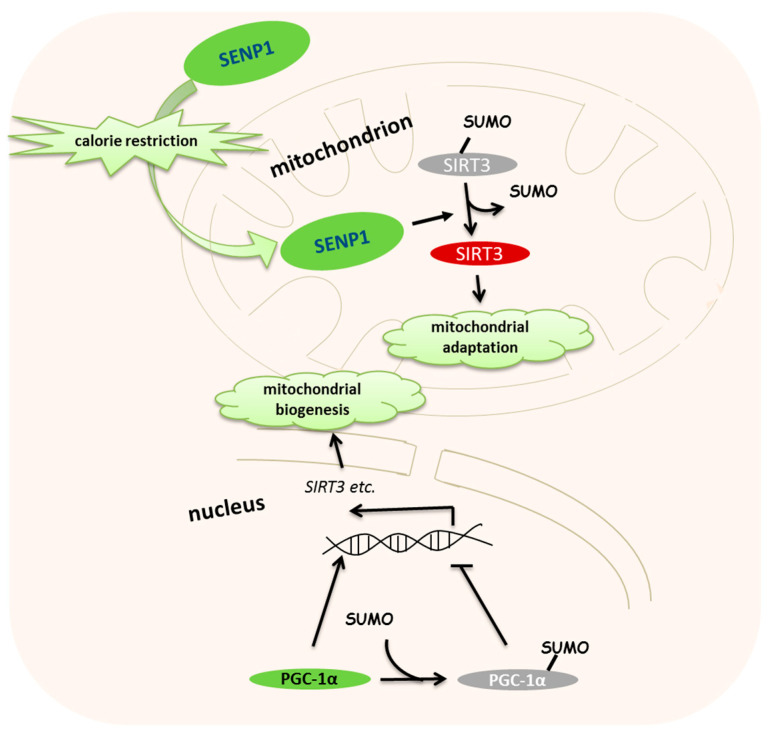
Small ubiquitin-related modifier (SUMO)ylation regulates mitochondrial biogenesis and metabolism upon stress. Upon mitochondrial stress, SUMO specific protease 1 (SENP1) translocates into the mitochondrial matrix and deSUMOylates NAD^+^-dependent deacetylase sirtuin 3 (SIRT3) immediately. Activated SIRT3 by deSUMOylation satisfies the response to the mitochondrial stress in a short time. Later, peroxisome proliferator-activated receptor γ (PPARγ) coactivator 1α (PGC-1α) in cooperation with the other transcriptional regulators increases mitochondrial protein-encoded gene expression, including *SIRT3*. Accumulated SIRT3 enhances the mitochondrial adaptation.

As a master regulator of mitochondrial biogenesis, peroxisome proliferator-activated receptor γ (PPARγ) coactivator 1α (PGC-1α) can active the expression of transcription factors and then regulate mitochondrial biogenesis and respiration [32]. Accumulated evidence shows that nuclear respiratory factor 1 (NRF-1), estrogen-related receptor (ERR) and peroxisome proliferator-activated receptor γ (PPARγ) are closely related to PGC-1α activation [33,34,35]. PGC-1α can be SUMOylated at K183 (mouse), and SUMOylation negatively regulates its PPARγ- and ERRγ-dependent transcription function [36,37,38] (Figure 2). More importantly, SENP1-mediated PGC-1α deSUMOylation enhances mitochondrial biogenesis and function [38]. However, the genetic induction of SENP1 enhances PGC-1α transcription, causing cardiac mitochondrial dysfunction via the deSUMOylation of myocyte enhancer factor-2C (MEF-2C) [37]. In response to oxidative stress, PGC-1α relies on the transcription factor NRF-1, ERR and PPAR to control the nuclear transcription of genes encoding mitochondrial proteins [39,40,41]. Suppression of mitochondrial biogenesis by the SUMOylation of PGC-1α may reduce the parkin expression to cause mitochondrial swelling in Parkinson’s disease [42].

## 3. Roles of SUMOylation in Calorie Restriction-Induced Mitochondrial Metabolism

Mitochondria have been considered as cellular power plants that answer the challenges from various nutrient stresses. As with other post-translational protein modifications, SUMOylation is a transient and efficient way for the cell to answer nutrient stimuli. Upon fasting, SENP1 accumulates in the mitochondrial matrix and deSUMOylates nicotinamide adenine dinucleotide^+^ (NAD^+^)-dependent deacetylase SIRT3 within the first hour [24] (Figure 2). Then, the deSUMOylation of SIRT3 releases its deacetylase activity and enhances its substrate activity. SIRT3 is a major mitochondrial deacetylase with a broad range of substrates. It is able to deacetylate mitochondrial complex I and II to activate the electron transport chain and enhance oxidase phosphorylation [43]. Upon fasting, SIRT3 can induce fatty acid oxidation in hepatocytes via the deacetylation of long-chain acyl co-enzyme A dehydrogenase 2 (LCAD) [44]. SIRT3 also targets the mitochondrial key metabolic enzymes isocitrate dehydrogenase 2 (IDH2) and superoxide dismutase 2 (SOD2) [45,46,47], which function in part to maintain reactive oxygen species (ROS) homeostasis. SIRT3 deficiency increases ROS levels and hypoxia-inducible factor-1α (HIF-1α) stabilization, resulting in numerous age-related pathologies, such as hearing loss and tumorigenesis [47,48,49]. A comparison between SIRT3 wildtype (WT) and knockout (KO) mice revealed that SIRT3 promotes the urea cycle and amino acid catabolism during calorie restriction [50]. Acetyl-CoA synthase 2 (AceCS2) and 3-hydroxy-3-methylglutaryl CoA synthase 2 (HMGCS2) are SIRT3 target enzymes and are used to initiate Krebs cycle oxidation and ketone body synthesis, respectively [51]. Therefore, stress-induced deSUMOylation activates SIRT3 and reprograms the mitochondrial metabolism to improve mitochondrial adaptation. Based on this molecular mechanism, Sirt3 SUMOylation-deficient (Sirt3 K223R) mice were constructed to mimic the activated Senp1–Sirt3 signaling status. Finally, Sirt3 K223R mice exhibit high-fat-diet (HFD)-induced obesity resistance [24]. Since the SENP1–SIRT3 axis is proposed to play a mitochondrial stress modulator, there are a large number of questions waiting to be investigated including its roles in life span extension, blood glucose control and immune systems response.

## 4. Roles of SUMOylation in Oxygen-Induced Mitochondrial Dysfunction

When cells are exposed to a low oxygen, mitochondria readily induce a series of adaptive signals for cell survival. such as AMP-activated protein kinase (AMPK) pathway activation, SIRT3 substrates activation, etc. [52,53]. SUMOylation is involved in these signaling transductions [24,54,55,56], and the expression of SUMO1 has been shown to increase upon hypoxia [31]. Hypoxia-induced mitochondrial dysfunction is associated with type 2 diabetes, Alzheimer’s disease, cardiac ischemia/reperfusion injury, tissue inflammation and cancer [57,58,59,60,61]. Under hypoxia, less efficient anaerobic glycolysis is utilized to consume glucose and produce ATP and lactate. The alteration of the energy metabolism causes a decreased mitochondrial size, reduced mitochondrial respiration rate and altered mitochondrial morphology [62].

**Figure 3:**
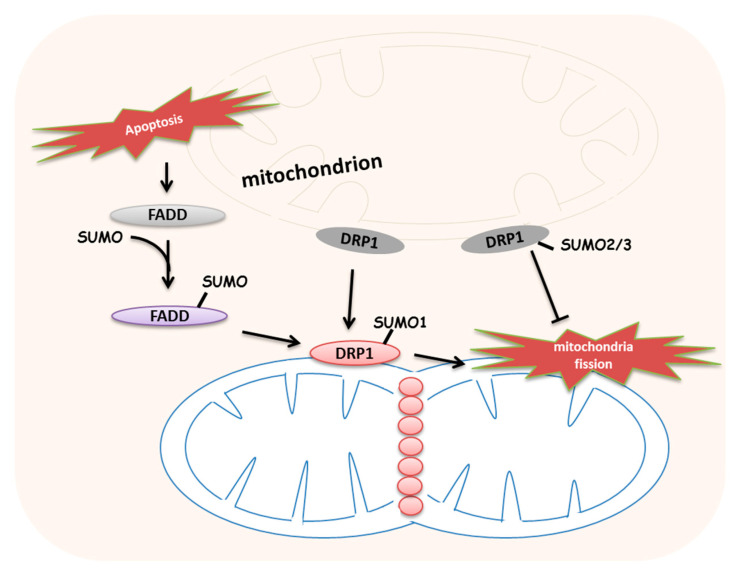
Small ubiquitin-related modifier (SUMO)ylation regulates mitochondrial morphology upon stress. Upon mitochondrial stress, SUMO1-modified dynamin-related protein 1 (DRP1) is protected from degradation and promotes mitochondrial fragmentation. However, SUMO2/3-conjugated DRP1 inhibits mitochondrial fission and cytochrome c release, and SUMOylated Fas-associated protein with death domain (FADD) promotes DRP1; DRP1-mediated mitochondrial fragmentation during apoptosis and necroptosis.

As storage tanks of calcium ions, mitochondria can absorb calcium ions and hold them until they are needed at a very fast rate. In response to the oxygen stress, mitochondria promote or prevent apoptosis to determine the cellular fate via buffering the cytosolic calcium. Interactively, excessive calcium influx and the intracellular accumulation of calcium commonly occur, affecting mitochondrial functions. Fas-associated protein with death domain (FADD) has been considered to participate in apoptosis or in necroptosis as a negative regulator after its ubiquitination [63]. A recent study has demonstrated that FADD was SUMOylated in the mitochondrion-rich fraction of mammalian cells after 24 h of hypoxia [64]. Reduced intracellular calcium ions and reoxygenation can prevent hypoxia-induced FADD SUMOylation [64]. SUMOylated FADD can promote dynamin-related protein 1 (DRP1)-mediated mitochondrial fragmentation during apoptosis and necroptosis [64] **(Figure 3**), which may indicate that SUMOylated FADD improves the mitochondrial calcium bank function upon hypoxia-induced cell death.

Processed mitochondrial redox protein thioredoxin 2 (Trx2) harbors anti-oxidant and anti-senescence activities [65], and excess ROS can interrupt the interaction between SUMOylated α-mitochondrial processing peptidase (MPP) and the SUMO interaction motif (SIM) of mitochondrial redox protein thioredoxin 2 (Trx2) to block Trx2 processing [66]. This is also regarded as a critical mechanism causing senescence-associated cardiovascular diseases.

## 5. Roles of SUMOylation in Stress-Induced Mitochondrial Dynamics and Mitophagy

Mitochondrial dynamics is a switch between fusion and fission, the alternation generally induced by the stress. In response to stress, the signal proteins distribute on the mitochondrial membranes. Outer membrane proteins mitofusin 1 and mitofusin 2 mediate the fusion of two mitochondrial outer membranes, while the inner membrane protein optic atrophy 1 (OPA1) regulates the inner membrane fusion [67]. Mitochondrial fission is a process whereby mitochondrial tubules undergo fragmentation and compartmentalization into daughter tubules; the outer membrane protein dynamin-related protein (DRP1) is the major regulator of this process [67]. SUMO1 has been found to be closely associated to the site of mitochondrial fission and colocalizes with endogenous DRP1 [68]. SUMO1 overexpression protects DRP1 from degradation and dramatically increases the level of mitochondrial fragmentation [68,69] (Figure 3). Evidence shows that the overexpression of SENP5 can prevent this fragmentation [70]. The deprivation of oxygen and glucose degrades SENP3 and increases SUMO-2/3 conjugation, especially on DRP1 SUMOylation to inhibit mitochondrial fragmentation and cytochrome c release [71] (Figure 3). An apoptosis-promoting protein, Bax/Bak, promotes DRP1 SUMOylation and enhances the permeability of the outer mitochondrial membrane [72].

On the other hand, a study shows that mitochondrial-anchored protein ligase (MAPL) functions as the first identified mitochondrial outer membrane SUMO E3 ligase to stimulate DRP1 SUMOylation and mitochondrial fission [21,70], and MAPL is also downstream of Bax/Bak [22]. The knockdown of MAPL in HeLa cells illustrates a down-regulation of mitochondrial global SUMOylation levels [21], which may suggest that MAPL is an essential ligase for the mitochondrial SUMOylation. A further study illustrated that MAPL-induced DRP1 SUMOylation at the ER/mitochondria contact sites is required for mitochondrial constriction, calcium transfer, cristae remodeling and even programmed cell death [22].

Phosphatase and tensin homolog (PTEN)-induced kinase 1(PINK1)-associated parkin is critical for mitochondrial dynamics, mitochondrial transport and mitophagy [73]. Parkin can interact with SUMO1 to induce self-ubiquitination and -translocation into the nucleus [74]. PINK1-dependent parkin induces the proteasomal degradation of mitochondrial proteins and mitophagy [75]. Thus, a SUMO1-induced lower parkin level increases substrate degradation and suppresses mitochondrial biogenesis through the accumulation of parkin interacting substrate [76,77].

## 6. Roles of SUMOylation in Mitochondrial Stress-Induced UPR^mt^

A number of senescence-associated proteins can be SUMOylated. The senescence induces mitochondrial unfolded protein response (UPR^mt^) accompanied with the repair of the mitochondrial respiration and mitochondria–nucleus crosstalk [78,79]. UPR^mt^ reprograms metabolism, initiates the innate immune response and even extends the lifespan [80,81,82]. Upon stress, mitochondrial import efficiency is impaired and the UPR^mt^ transcriptional factors ATFS-1(activating transcription factor associated with stress-1) and DVE-1 (homeobox domain-containing protein) accumulate in nuclei [83,84]. ATFS-1 regulates half of the mitochondrial stress response genes such as mitochondrial-specific chaperones and immune response and glycolysis-related genes [80,83]. Another transcriptional factor DVE-1 binds to the H3K9me2-less chromatin and controls the mitochondrial stress response genes [84]. A recent study found that both DVE-1 and ATFS-1 were SUMOylated proteins in *Caenorhabditis elegans* [85]. ULP-4 is a SUMO-specific protease in *C. elegans*. Furthermore, Gao et al.’s study identified that ULP4 deSUMOylates ATFS-1 at the K326 residue and DVE-1 at the K327 residue [85]. During this process, deSUMOylation affects the stability and transcriptional activity of ATFS-1 and alters the subcellular localization of DVE-1 from the cytosol to nucleus [85]. ATFS-1 and DVE-1 SUMOylation deficiency triggers UPR^mt^ to initiate the innate immunity response and prolong the lifespan [85]. DVE-1 shares homologs with the human SATB (special AT-rich sequence-binding) class of proteins that also function in chromatin remodeling and transcription, and SATB1 and SATB2 are also SUMOylated proteins [86,87]. SUMOylated SATB1 can anchor to the promyelocytic leukemia (PML) nuclear bodies where it undergoes caspase cleavage [87], and SATB1 in the nucleus can bind H3K9 and H3K14 sites for their acetylation and methylation switching [88]. SUMOylated SATB2 can translocate to the nuclear periphery to regulate immunoglobulin μ gene expression [86]. However, there is no further evidence for SATB in UPR^mt^. Mammalian ATF5 has homologs with ATFS-1 [89]; whether ATF5 can be SUMOylated or not is still unknown.

## 7. Roles of SUMOylation in Mitochondrial Stress-Induced Diseases

**Figure 4:**
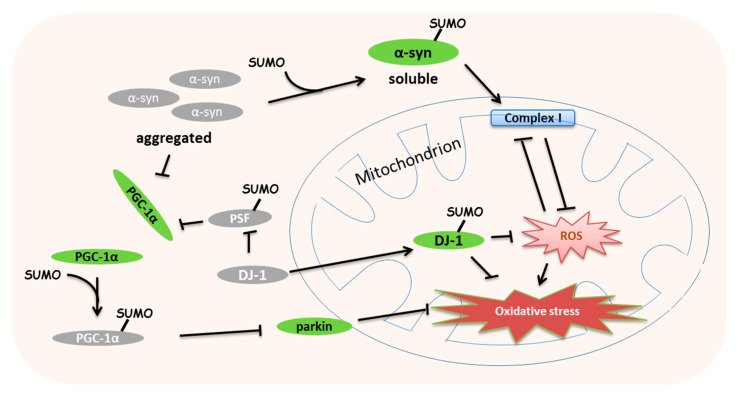
Small ubiquitin-related modifier (SUMO)ylation regulates the oxidative stress in neurodegeneration. Upon mitochondrial stress, SUMOylated α-synuclein (α-syn) increases its solubility, and then protects the mitochondrial complex I to reduce reactive oxygen species (ROS). The SUMOylation of Parkinson’s desease protein 7 (DJ-1) enhances the mitochondrial response to the oxidative stress. DJ-1 decreases the SUMOylated protein-associated splicing factor (PSF) to improve peroxisome proliferator-activated receptor γ (PPARγ) coactivator 1α (PGC-1α)-mediated mitochondrial biogenesis. SUMOylated PGC-1α reduces parkin expression and suppresses mitochondrial biogenesis.

Mitochondrial stress also leads to neuronal dysfunction. During this process, SUMOylation is essential for synaptic transmission, plasticity and neuroprotection [90]. Decreased solubility of α-synuclein (α-syn) is a common feature of Parkinson’s disease (PD). Mutations of α-syn have been considered to be associated with classic late-onset PD, Lewis bodies’ formation and even dementia [91]. Later, a series of studies demonstrated that α-syn can be SUMOylated at K96 and K102 in the halo of Lewis bodies and decreased α-syn SUMOylation increases its aggregation and toxicity [92,93] (Figure 4). However, a co-expression assay in COS-7 cells identified that SUMOylation increases α-syn aggregation [94]. Mitochondrial stress is one of the reasons for the accumulation of α-syn [95], and α-syn accumulation induces complex I impairment and elevated ROS levels [96](Figure 4). UV irradiation increases the SUMOylation of PD (autosomal recessive, early onset) 7 (DJ-1) at K130 and decreases its solubility in the cell line [97,98,99,100]. The L166P mutation of DJ-1 found in PD patients can be SUMOylated abnormally and loses its response to mitochondrial stress [97]. DJ-1 can decrease the global SUMOylation level, especially the SUMOylation of pyrimidine tract-binding protein-associated splicing factor (PSF), to increase the expression of tyrosine hydroxylase, while abnormal SUMOylated DJ-1 causes impaired dopamine synthesis and PD development [101](Figure 4). In addition, α-syn may reduce PGC-1α to induce dopaminergic neuronal death, and α-syn mutation-induced mitochondrial loss can be protected by the restoration of PGC-1α [102,103]. Interestingly, SUMOylated PSF promote the interaction with PGC-1α to repress its transcriptional activity [104]; in harmony with this, SUMOylation also suppresses the activity of PGC-1α [38] (Figure 3). There are no fragmented mitochondria found in parkin mutation fibroblasts from PD patients [105]. Accordingly, this may suggest that mitochondrial dysfunction is triggered by oxidative stress in PD patients.

Amyotrophic lateral sclerosis (ALS) associates with the motor neurons dysfunction [106]. Superoxide dismutase 1 (SOD1) mutations have been considered as responsible signals in familial ALS patients, and the mutations cause SOD1 instability and aggregation [107]. SUMO modification can increase the aggregation of SOD1 mutations [108,109], while SENP can alleviate this accumulation [110]. The SOD1 mutations increase the free radicals in the inter-membrane space of mitochondria and cause the axonal transport dysfunction [111]. This suggests that SENP is a potential therapeutic target for familial ALS.

Alzheimer’s disease (AD) is characterized by an aberrant aggregation of amyloid-β (Aβ) and the microtubule-associated protein tau proteins. Interestingly, the SUMOylated proteins and SUMOylation regulates their abnormal aggregation [112,113]. As known, AD is an age-associated disease with mitochondrial dysfunction [114]. In the near future, combining the SUMOylation-mediated protein aggregation with the improvement of mitochondrial adaptation may be a breakthrough point to treat AD.

In the patients with type 2 diabetes, the insulin secretion function of β cells from the pancreatic islets of Langerhans is impaired. In agreement with the isocitrate export of mitochondria, Ferdaoussi et al.’s study found that SENP1 coupled with the redox state in β cells and amplified the glucose-dependent insulin exocytosis to repair glucose tolerance [115]. This investigation indicates that type 2 diabetes can be triggered by mitochondrial metabolites, and several SUMOylated proteins may be involved in this glucose- and oxidative stress-dependent protective mechanism.

Generally, the genotoxic stresses, such as chemotherapeutic agents, ionizing radiation, and UV exposure etc., can generate DNA damage to cause tumorigenesis. In response to these genotoxic stresses, SUMOylation or deSUMOylation occurs to mediate the cellular status. Until recently, many oncogenes and tumor suppressors have been reported to be SUMOylated. For instance, SUMOylated tumor suppressor 53BP1 can regulate the genome maintenance [116]. However, little is known regarding whether SUMOylation mediates the alteration of mitochondrial function or morphology during the proliferation, growth and apoptosis of tumor cells.

## 8. Conclusions and Further Perspective

More than 20 years have passed since the discovery of SUMO; in that time, SUMO has been determined to be a key regulator of various cellular events. Because of its ubiquitous nature, many questions are left to be addressed. Currently, we know that many SUMOylated proteins can respond to mitochondrial stress, as depicted in Table 1, but it is as yet unknown how the SUMOylation level of specific metabolic pathways changes upon different stresses. We also still need to explore whether SUMOylation affects mitochondrial functions via the key enzyme of the corresponding pathway, and the network by which the crosstalk of the different post translational modifications occurs in mitochondria has yet to be clarified. Fortunately, increasing numbers of studies are providing clues for further investigations.

Calorie restriction promotes a lower SUMOylation level of mitochondria, indicating that nutrient stress is a trigger that alters the SUMOylation level [24]. Different stresses may be associated with different SUMOylation patterns. To some extent, the SUMOylation level of mitochondria reflects mitochondrial adaptation. SENP1 has been found to be a nutrient sensor which translocates into mitochondria after a short period of starvation. Accordingly, the accumulation level of SENP1 in mitochondria can be regarded as an indicator of the degree of mitochondrial stress response. The expression level of MAPL at the ER/mitochondria contact sites may reflect the degree of mitochondrial fragmentation [22,24]. However, the molecular mechanism of SENP1 translocation into mitochondrial is still unknown. Once SENP1 translocation is regulated, the SUMOylation level and metabolism of mitochondria can be further controlled.

Mitochondria are the most important regulators of energy and matter in cells. Nutrient uptake links the metabolism of carbohydrates, fats, and proteins by the mitochondria. These macromolecules are oxidized to acetyl CoA and are further catalyzed through eight enzymatic steps of the Krebs cycle. The oxidation of these organic macromolecules drives ATP synthesis via electron transport chain complexes. Fatty acids, which are the oxidation products of fats, can be finally β-oxidized to the acetyl-CoA that enters the Krebs cycle. Several key enzymes of the Krebs cycle, oxidative phosphorylation and β-oxidation have been predicted to be SUMOylated by comprehensive proteomics analysis [117]. Since SUMO, SAE1 and UBC9 are all detected in the mitochondrial matrix [24], the role of SUMOylation on mitochondrial metabolism—especially upon nutrient stresses—will be clarified soon.

It is worthwhile to note that the components of the SUMOylation system can be regulated by phosphorylation. Similar to SUMOylation, there are some proteins which phosphorylation associates with mitochondrial oxidative stress. Accumulating evidence shows that there is an inter-connection between SUMOylation and phosphorylation [118,119,120,121]. Recent studies have shown that obesity and aging decrease SIRT1-mediated SIRT3 deacetylation and reduce SIRT3 stability and activity [122]. Nutrient stress can also alter SIRT1 expression to affect mitochondrial biogenesis via the deacetylation of PGC-1α [123]. In response to mitochondrial stress, the regulating effects of deacetylation are consistent with deSUMOylation in mitochondria. Accordingly, the development of synergistic agonists for SENP1 and SIRT1 is expected to improve mitochondrial health.

## Figures and Tables

**Table 1:** Summary of SUMOylated proteins that function in response to mitochondrial stress.

Proteins	SUMOylation Site	SUMO-Specific Protease	Function of SUMOylation	References
PCG-1α	K183 (mouse)	SENP1, SENP2	SUMOylation suppresses its function of transcriptional biogenesis	Rytinki and Palvimo, 2009 [36]
FADD	K120/K125/K149 (human)	-	SUMOylations promotes mitochondrial-associated necrosis	Choi et al., 2017 [64]
DRP1	K532/K535/K558/ K568/K594/K597/ K606/K608 (human)	SENP3	SUMO2/3 modification suppresses mitochondrial fragmentation	Figueroa-Romero et al., 2019 [69]; Zunino et al., 2009 [70]; Guo et al., 2013 [71]
		SENP5	SUMO1 modification promotes mitochondrial fragmentation	
SNCA (α-synuclein)	K96/K102 (human)	-	SUMOylation inhibits α-syn accumulation to protect the mitochondrial complex I	Krumova et al., 2011 [93]
DJ-1	K130 (human)	-	Abnormal SUMOylation lost the response to the mitochondrial stress	Shinbo et al., 2006 [97]
PSF	K338 (human)	SENP1	SUMOylation promotes the interaction with PGC-1α to repress mitochondrial biogenesis	Zhong and Xu, 2008 [104]
SIRT3	K228 (human) K223 (mouse)	SENP1	SUMOylation suppresses its function as an NAD^+^-dependent deacetylase	Wang et al., 2019 [24]
